# Safety of High-Dose Vitamin C in Non-Intensive Care Hospitalized Patients with COVID-19: An Open-Label Clinical Study

**DOI:** 10.3390/jcm13133987

**Published:** 2024-07-08

**Authors:** Salvatore Corrao, Massimo Raspanti, Federica Agugliaro, Francesco Gervasi, Francesca Di Bernardo, Giuseppe Natoli, Christiano Argano

**Affiliations:** 1 Department of Internal Medicine, National Relevance and High Specialization Hospital Trust ARNAS Civico, Di Cristina, Benfratelli, 90127 Palermo, Italy; federica.agugliaro@gmail.com (F.A.); peppenatoli@gmail.com (G.N.); chargano@yahoo.it (C.A.); 2 Department of Health Promotion Sciences, Maternal and Infant Care, Internal Medicine and Medical Specialties (PROMISE), University of Palermo, 90127 Palermo, Italy; 3 Cardiology and Intensive Care Unit, A. Aiello Hospital, 91026 Mazzara del Vallo, Italy; massimoraspanti1991@gmail.com; 4 Specialized Laboratory of Oncology, National Relevance and High Specialization Hospital Trust ARNAS Civico, Di Cristina, Benfratelli, 90127 Palermo, Italy; francesco.gervasi@arnascivico.it; 5 Department of Microbiology and Virology, National Relevance and High Specialization Hospital Trust ARNAS Civico, Di Cristina, Benfratelli, 90127 Palermo, Italy; francesca.dibernardo@arnascivico.it

**Keywords:** vitamin C, safety, length of hospital stay, in-hospital mortality, admission to ICU

## Abstract

**Background**: Vitamin C has been used as an antioxidant and has been proven effective in boosting immunity in different diseases, including coronavirus disease (COVID-19). An increasing awareness was directed to the role of intravenous vitamin C in COVID-19. **Methods**: In this study, we aimed to assess the safety of high-dose intravenous vitamin C added to the conventional regimens for patients with different stages of COVID-19. An open-label clinical trial was conducted on patients with COVID-19. One hundred four patients underwent high-dose intravenous administration of vitamin C (in addition to conventional therapy), precisely 10 g in 250 cc of saline solution in slow infusion (60 drops/min) for three consecutive days. At the same time, 42 patients took the standard-of-care therapy. **Results**: This study showed the safety of high-dose intravenous administration of vitamin C. No adverse reactions were found. When we evaluated the renal function indices and estimated the glomerular filtration rate (eGRF, calculated with the CKD-EPI Creatinine Equation) as the main side effect and contraindication related to chronic renal failure, no statistically significant differences between the two groups were found. High-dose vitamin C treatment was not associated with a statistically significant reduction in mortality and admission to the intensive care unit, even if the result was bound to the statistical significance. On the contrary, age was independently associated with admission to the intensive care unit and in-hospital mortality as well as noninvasive ventilation (N.I.V.) and continuous positive airway pressure (CPAP) (OR 2.17, 95% CI 1.41–3.35; OR 7.50, 95% CI 1.97–28.54; OR 8.84, 95% CI 2.62–29.88, respectively). When considering the length of hospital stay, treatment with high-dose vitamin C predicts shorter hospitalization (OR −4.95 CI −0.21–−9.69). **Conclusions**: Our findings showed that an intravenous high dose of vitamin C is configured as a safe and promising therapy for patients with moderate to severe COVID-19.

## 1. Introduction

It is four years since the World Health Organization declared COVID-19 a pandemic on 11 March 2020 [1] after the first pneumonia cases caused by severe acute respiratory syndrome coronavirus (SARS-CoV2) occurred in late December 2019 [2]. Globally, as of 3 March 2024, there have been 774,833,487 confirmed cases, and 7,036,994 deaths have been reported worldwide [3]. This pandemic notably challenged the healthcare system and medical care, highlighting the lack of preparation and inadequacy to manage this public health crisis and simultaneously provide general and specialized medical care [4]. Despite a more in-depth understanding of the pathophysiologic mechanisms behind COVID-19 [5,6] and the recent progress in managing and averting COVID-19 by utilizing vaccines and antiviral drugs, effective interventions have remained a significant challenge [7,8], especially in older people with comorbidities [9]. From the beginning of COVID-19, researchers have studied and used molecules and drugs with anti-inflammatory and antioxidant properties [9,10,11,12,13,14]. In this sense, an increasing interest was developed in utilizing vitamin C [15,16,17]. Vitamin C, also known as ascorbic acid, is a water-soluble vitamin that acts as a strong antioxidant and plays a role as a cofactor in various biosynthetic pathways in the immune system. It is an essential nutrient that the human body cannot produce on its own [18]. Its antioxidant properties stem from its ability to donate electrons, which helps protect molecules from oxidative damage.

For this reason, many studies have focused on the antioxidant properties of vitamins, particularly their potential anticancer and antiatherosclerosis properties [19]. In addition, vitamin C stimulates immunity through the raised activity of neutrophils by increasing chemotaxis and phagocytosis and in T-cell maturation, as well as through the mechanism of ascorbate-mediated enhancement of immune function [20,21,22]. Individuals with a deficiency in vitamin C face an increased likelihood of developing severe respiratory infections, such as pneumonia [23,24]. A meta-analysis has shown that oral vitamin C supplements can reduce the risk of pneumonia, especially in those with low dietary intakes [25]. On the contrary, a recent meta-analysis showed no reduction in mortality among people with community-acquired pneumonia [26]. Vitamin C deficiency can also elevate the risk of sepsis during pneumonia.

Moreover, among patients with a diagnosis of sepsis and clinically significant vitamin C deficiency, a more rapid progression of multiorgan failure was observed [27]. In this sense, contrasting data are available about the combination of thiamine, vitamin C, and hydrocortisone for the treatment of septic shock; in fact, Marik and colleagues [28] discovered that early intravenous administration of vitamin C, complemented with thiamine and corticosteroids, is effective in preventing progressive organ dysfunction. Litwak et al. showed that triple therapy did not improve hospital or ICU mortality in patients with septic shock [29]. Various clinical studies evaluating high-dose intravenous vitamin C administration in patients with severe sepsis, acute lung injury, and acute respiratory distress syndrome admitted to intensive care units have shown mixed results regarding laboratory and clinical outcomes [30,31,32]. A systematic review and meta-analysis of intravenous vitamin C therapy in critically ill patients showed favorable results regarding decreased mechanical ventilation support to reduce overall mortality [33]. A recent systematic review and meta-analysis showed no significant effect on infection episodes, length of stay in the hospital or ICU, or duration of mechanical ventilation of administration of intravenous vitamin C treatment in critically ill patients. However, the study did indicate a mild tendency towards reducing mortality [34]. However, a recent meta-analysis revealed a decrease in the duration of mechanical ventilation [35].

In addition, vitamin C inhibits viral growth, stimulates interferon production, and enhances lung epithelial cells’ antiviral activity [36,37]. The antiviral properties of vitamin C are believed to be particularly beneficial in treating COVID-19. However, how vitamin C is administered is crucial for both its effectiveness and safety. It is crucial to remember that while injections and infusions provide the highest dose of vitamin C, they also pose a greater risk of overdose and renal side effects. This is because they bypass the natural limits of intestinal transporters. It is important to note that higher doses of vitamin C administered via infusion may lead to more serious side effects, such as severe kidney injury [38].

Since the outbreak of the SARS-CoV-2 pandemic, the safety and effectiveness of high-dose vitamin C have been the focus of scientific research. Numerous studies have tried to assess the effectiveness of vitamin C in preventing COVID-19 and its capacity to prevent the progression to more severe diseases. Additionally, the impact of high doses of vitamin C on critically ill patients was evaluated [15,39,40]. Given this background, this study aimed at evaluating the safety and, secondly, the effectiveness of the administration of a high dose of vitamin C (10 g for three consecutive days) in addition to standard-of-care therapy in a cohort of hospitalized patients with COVID-19 in the non-intensive care ward, as well as the effect on mortality, length of hospital stays, and admission to the intensive care unit.

## 2. Materials and Methods

From 30 March 2020 to 1 April 2021, 146 patients with confirmed COVID-19 (RT-PCR positive for SARS-CoV-2 and presence of typical radiological signs) who were admitted to the COVID Unit of the Department of Internal Medicine of the National Relevance and High Specialization Hospital Trust ARNAS Civico, Di Cristina, Benfratelli, Italy, were consecutively enrolled in this longitudinal study. The patients admitted to the Department of Internal Medicine all provided written informed consent upon admission. Eligible patients were adults with proven COVID-19 admitted to the hospital. There are no exclusion criteria other than the lack of informed consent signature. The Ethics Committee has approved the conduct of the study at our institution. The approval number assigned to this study is 3143. Of these patients, 104 underwent high-dose intravenous administration of vitamin C (in addition to conventional therapy); precisely, 10 g in 250 cc of saline solution in slow infusion (60 drops/min) for three consecutive days, according to the therapeutic protocol “Use of Ascorbic Acid in patients with COVID-19” developed by our center and registered in March 2020 on the platform Clinicaltrials.gov (accessed on 14 January 2024). NCT04323514 [41]. The other 42 patients underwent the standard of care therapy. Variables taken into consideration for all patients at the time of admission included age, sex, pre-existing comorbidities, whether or not oxygen therapy was required, the method used [nasal cannula (N.C.), Venturi Mask (MV), Continuous Positive Airway Pressure (CPAP), or noninvasive ventilation (N.I.V.)], and levels of inflammatory markers, such as lactate dehydrogenase (L.D.H.), procalcitonin (P.C.T.), C-reactive protein (C.R.P.), D-Dimer (D-D), and interleukin-6 (IL-6).

### Statistical Analysis

Quantitative variables are summarized as mean (95% confidence intervals), medians (interquartile range: first–third), and categorical variables, reported as percentages. The safety of vitamin C administration represented the primary outcome. The secondary outcome included mortality and length of hospital stay. In order to establish the safety of administering high doses of intravenous ascorbic acid, all adverse events were recorded during and after administration. Furthermore, a patient’s control group was consecutively recruited to use multivariable logistic regression to study a possible association with the hospital mortality of treated subjects. The following clinically relevant variables were considered to demonstrate safety or lack of safety: sex, age, use of noninvasive ventilation methods, obesity, and diabetes. The quantitative variables were compared using the non-parametric Mann–Whitney U test. A multivariate regression analysis investigated the relationship between variables and in-hospital mortality, admission to the intensive care unit, and length of hospital stays.

The Hosmer–Lemeshow methodology [42] was used to select variables for analysis [39]. After univariate analysis, only variables with a *p*-value less than 0.20 were included in the final model. The variables were excluded through a backward process until each variable reached a significance level of *p* < 0.20. The Hosmer–Lemeshow test was used to measure how well the model fits the data without the researcher having to choose any variables for the multivariate model. Statistical significance was set at a two-tailed *p*-value of less than 0.05. Stata Statistical Software 2017 (StataCorp, College Station, TX, USA) was used for database management and analyses.

## 3. Results

During the recruitment period, 146 hospitalized patients were examined. Among these, 59.6% were male, with a median age of 64.3 years. Table 1 shows the clinical characteristics of the study population.

In total, 80.4% of all patients had at least one pre-existing condition at admission. Disease distribution showed that arterial hypertension, obesity (B.M.I. ≥ 30 kg/m^2^), and diabetes mellitus were the most represented comorbidities in patients with COVID-19 (70%, 40.8%, and 35.6%, respectively). Furthermore, almost a third of patients reported a previous major ischemic event (myocardial infarction and stroke). For approximately two-thirds of patients, oxygen supportive therapy was necessary on admission or during a hospital stay, with over a quarter of subjects requiring ventilation with continuous positive airway pressure (CPAP) or noninvasive ventilation with pressure support ventilation (NIV/ST). Due to severe hypoxemia, 8.3% were admitted to intensive care. Death occurred in 10.2% of patients. The average length of hospital stay was 19 days.

The clinical characteristics of patients with COVID-19 who underwent intravenous vitamin C and conventional therapy are shown in Table 2. No significant differences in the main variables were found between the two groups. A more significant percentage of patients not undergoing IHDVC required venturi mask and CPAP in comparison with people undergoing IHDVC (30.9% vs. 23.1% and 23.8% vs. 12.5%, respectively), while the percentage of patients that require NIV S/T was higher in people who received IHDVC (12.5% vs. 7.1%). Almost 10 percent of patients taking vitamin C were admitted to the intensive care unit (9.7% vs. 5.0%). It is worth outlining that a shorter hospital length of stay and mortality were present in patients who underwent high doses of vitamin C compared to those who took conventional therapy (18.0% vs. 24.0% and 8.6% vs. 14.3%, respectively). 

Table 3 shows the laboratory characteristics of patients who underwent intravenous vitamin C and conventional therapy. This table represents the median percentage change (Q1–Q3) between the value recorded at discharge and the value recorded at admission for patients who took intravenous high doses of vitamin C and those who did not.

A significant increase in the median percentage change of neutrophils was found in patients who took an intravenous high dose of vitamin C compared to those who took conventional therapy (*p* = 0.0126). Compared to the control group, no significant changes were highlighted in other laboratory variables, such as C-reactive protein and procalcitonin. During the observation, there were no differences in terms of adverse drug events between the intervention group and the control group. We did not record any adverse events. Since the main side effect and the main contraindication are related to chronic renal failure, we evaluated the renal function indices and estimated the glomerular filtration rate (eGRF) (calculated with the CKD-EPI Creatinine Equation). No statistically significant differences were found between the two groups. Furthermore, no allergic reactions occurred.

**Figure 1:**
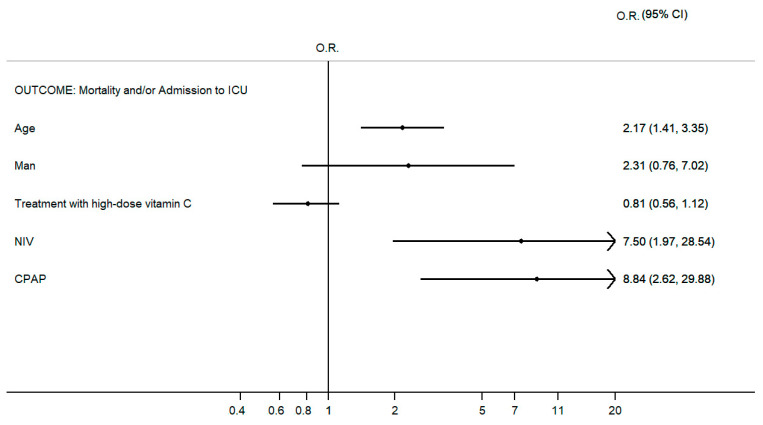
Multivariable logistic regression according to composite outcome mortality and/or admission to the intensive care unit (*p* < 0.0001; pseudo R^2^ 31.3%). Only the final model is shown according to the Hosmer–Lemeshow methodology for selecting variables; see the statistical analysis section.

As illustrated in Figure 1, the high-dose vitamin C treatment did not result in a statistically significant decrease in mortality and admission to the intensive care unit, even if the result bordered on statistical significance. On the contrary, age was independently associated with in-hospital mortality and admission to the intensive care unit, as well as N.I.V. and CPAP (OR 2.17, 95% CI 1.41–3.35; OR 7.50, 95% CI 1.97–28.54; OR 8.84, 95% CI 2.62–29.88, respectively). When we consider the length of hospital stay (Table 4), treatment with high-dose vitamin C represents a predictor of shorter length of hospitalization. Although length of hospital stay was not statistically significantly different between the two groups, a statistically significant difference was found when correcting the confounding variables. This difference was not only statistically significant but also clinically relevant. The data mentioned above allowed us to demonstrate an excellent safety profile of vitamin C in the short term.

## 4. Discussion

### 4.1. Vitamin C (Ascorbic Acid)

Vitamin C (ascorbic acid) is a water-soluble vitamin that potentially benefits patients with different disease severities. It is a “scavenger” molecule (a substance capable of transforming oxygen radicals into non-radical compounds, lacking reactivity and therefore toxicity) that has anti-inflammatory properties, influences vascular integrity and cellular immunity, works as a cofactor in the endogenous generation of catecholamines, and has been studied in many disease states (predominantly inflammatory states), including COVID-19 [31]. Vitamin C is crucial in forming and depositing type IV collagen in the basement membrane. It also promotes endothelial proliferation, inhibits apoptosis, and preserves endothelial cell-derived nitric oxide to help regulate blood flow [43].

Vitamin C plays a vital role in the functioning and regulation of the immune system. Leukemic cells and neutrophils accumulate vitamin C intracellularly, which depends on its availability in plasma. In neutrophils, vitamin C affects the phagocytosis of microorganisms and chemotaxis. In addition, vitamin C protects neutrophils and phagocytes from damage following an oxidative burst through its antioxidant and scavenging capacity and activates a caspase-dependent cascade that induces programmed apoptosis and inhibits necrosis [44,45].

A similar protective effect against oxidative stress has also been observed in lymphocytes. Vitamin C’s impact on controlling inflammation includes modifying nuclear transcription factor kappa B (NFkB) and reducing inflammatory cytokine production [46]. A recent systematic review and meta-analysis showed that the use of vitamin C reduces hospital mortality in patients with COVID-19 [17].

### 4.2. Role of Intravenous Vitamin C in Hospitalized Patients with COVID-19

The World Health Organization has identified vitamin C as a potential adjunctive therapy for patients with critical COVID-19 [47]. Several trials have indicated some potentially beneficial effects of intravenous vitamin C in severe COVID-19 [48,49]. In a pilot study in China, 56 adults in the ICU with COVID-19 were given either vitamin C or a placebo. The study was stopped early due to decreased COVID-19 cases. The results showed no differences in mortality, mechanical ventilation duration, or SOFA score change between the groups. The study showed that the treatment arm had more significant respiratory function improvements than the placebo arm. The improvement was evaluated by calculating the ratio of partial blood pressure of oxygen to inspired oxygen fraction (PaO_2_/FiO_2_) from day 1 to day 7. The change in the treatment arm was +20.0, while in the placebo arm, it was −51.9. The difference between the two arms was statistically significant (*p* = 0.04) [48]. Khumari found that intravenous vitamin C can significantly improve the clinical symptoms of patients affected by COVID-19. Although it may not impact mortality and the need for mechanical ventilation, patients who received vitamin C became asymptomatic earlier (7.1 days vs. 9.6 days; *p* < 0.0001) and had a shorter duration of hospitalization (8.1 days versus 10.7 days; *p* < 0.0001) compared to those who received standard therapy alone. There were no significant differences in mortality and the need for mechanical ventilation [49]. Li and colleagues, on the contrary, demonstrated that adjunctive IV vitamin C may not reduce mortality, vasopressor requirements, SOFA scores, or ventilator settings in critically ill COVID-19 patients [50]

### 4.3. The Immune System

The immune system is the body’s foremost defense against infectious agents. Throughout biological evolution, it has developed two central and different defense systems: non-specific (or innate) immunity and specific (or adaptive) immunity [51].

Innate and adaptive immune responses interact to create immune defenses [52]. The innate immune response occurs immediately after infection and is usually involved in virus elimination but with reduced antiviral capacity. Adaptive immunity is essential in complete virus elimination [52]; this immune pathway is activated 4–7 days after infection. The innate immune response is enhanced if the adaptive antiviral response fails to suppress the virus in time. However, it cannot effectively eliminate the virus, leading to a systemic inflammatory response with uncontrolled release of inflammatory cytokines [53,54,55,56]. Elderly patients and patients with chronic diseases require more time to develop an adaptive or innate immune response due to progressive cellular aging. Such patients rely solely on an enhanced antiviral innate immune response in the early stages of infection and are at increased risk for cytokinin storms. Whether the enhanced immunity is due to ongoing viral replication or immunomodulatory dysregulation remains unclear [57]. Finally, the NLR family containing pyrin domain 3 (NLRP3) is the most recognizable inflammasome pattern in COVID-19 and includes most of the above-mentioned immune–inflammatory pathways [58]. NLRP3 is a critical component of innate immunity and promotes inflammation by producing IL-1β/18 and inducing pyroptosis. Later, IL-1β and IL-18 play a role in promoting the release of other NLRP3 cytokines, including IL-6 [59]. For example, a study demonstrated that activation of the NLRP3 inflammasome of S. suis results in the production of IL-1β, leading to a cytokine release syndrome [60]. Consequently, a positive correlation has been observed between IL-18, caspase-1, and other inflammatory markers like C-reactive protein, lactate dehydrogenase (L.D.H.), and IL-6, in association with inflammasome activation in COVID-19 [61], suggesting an essential role of the NLRP3 inflammasome in forming cytokine storms and the pathogenesis of COVID-19. Thus, controlling NLRP3 inflammasome activation is a potential therapeutic strategy for these conditions. Ascorbic acid, or vitamin C, has been studied for its effects on the NLRP3 inflammasome, given its roles in immune function, antioxidant defense, and inflammation modulation. The interaction between ascorbic acid and the NLRP3 inflammasome involves several mechanisms. One of the known triggers for NLRP3 inflammasome activation is oxidative stress, characterized by a derangement between the generation of reactive oxygen species (R.O.S.) and the body’s ability to detoxify these reactive products or repair the resulting damage. Ascorbic acid, with its potent antioxidant properties, can scavenge R.O.S., thereby reducing oxidative stress and potentially preventing the inappropriate activation of the NLRP3 inflammasome. In addition, ascorbic acid can modulate the production and activity of various inflammatory molecules and cytokines, some of which are involved in the activation pathway of the NLRP3 inflammasome. By influencing these pathways, ascorbic acid might indirectly impact the activation and function of the NLRP3 inflammasome. Finally, emerging evidence suggests that ascorbic acid may directly inhibit the NLRP3 inflammasome. This direct inhibition can occur through the modulation of mitochondrial integrity, reduction of mitochondrial R.O.S., and preservation of cellular homeostasis, all of which are critical factors in activating the NLRP3 inflammasome. By stabilizing mitochondria and reducing mitochondrial R.O.S., ascorbic acid may prevent the assembly and activation of the NLRP3 inflammasome complex. However, while preclinical studies have shown promising results, clinical evidence supporting the efficacy of ascorbic acid in modulating the NLRP3 inflammasome in humans is still limited. Further research, including rigorous clinical trials, is imperative for a comprehensive understanding of the potential of ascorbic acid in managing conditions associated with NLRP3 inflammasome dysregulation. Additionally, the optimal dosing, safety, and long-term effects of ascorbic acid supplementation for these specific purposes need to be thoroughly evaluated.

### 4.4. Role of the Immune System in COVID-19

Patients with COVID-19 often exhibit mild neutrophilia and T-cell lymphopenia, leading to an increased neutrophil-to-lymphocyte ratio (NLR), which serves as a valuable prognostic marker for the severity of COVID-19 [62]. Other leukocyte subpopulations also undergo characteristic fluctuations and modifications, although these are more heterogeneous.

During an infection, viral PAMPs are detected by Toll-like receptors (TLRs), which then trigger intracellular signaling cascades. These cascades activate transcription factors, such as nuclear factor-kappa B (NF-κB) and interferon regulatory factors, leading to the production of type I interferons (IFNs) and proinflammatory cytokines. A proper IFN response usually leads to an antiviral immune state in infected cells, limiting viral replication and inducing apoptosis to protect the host from viral spread. However, certain SARS-CoV-2 proteins (e.g., open reading frames 6 and 3b) have been found to suppress type I IFN production and the antiviral signal [63]. The initial delay of the IFN response is followed by uncontrolled viral replication and spread in the infected host, contributing to a final surge in IFN that may worsen hyperinflammation in the progression to severe disease [64]. Another important component of the innate immune response is the complement system, which serves as a rapid immune surveillance system against invading pathogens, connecting innate and adaptive immunity.

In COVID-19 infection, excessive complement activation results in inflammation, endothelial cell dysfunction, and intravascular coagulation [65]. Data indicated that in patients with moderate to severe COVID-19 infection, there is an accumulation of Natural Killer (NK) cells in the lungs. In contrast, the number of the same cells decreases in the peripheral blood. Current evidence on the immune function of NK cells in COVID-19 is contradictory. While some studies have reported signs of hyporesponsiveness and depletion of NK cells in the blood of patients with COVID-19, others recorded a marked activation of these cells [66]. Finally, the innate immune system interacts with coagulation—a process known as “immuno-thrombosis”—which is thought to be dysregulated in severe COVID-19 infection, probably because of increased tissue factor expression (TF). This action, in turn, triggers the extrinsic pathway of coagulation [57]. Neutrophils, when activated, release neutrophil extracellular traps (NETs) which are composed of neutrophil-derived DNA and acetylated histones. These NETs function to trap and kill pathogens, but they can also trigger a strong procoagulant response. They can promote the activation of the intrinsic coagulation pathway through the activation of factor XII. Furthermore, they can bind TF to activate the extrinsic coagulation pathway or form aggregates with platelets, influencing the severity of the disease [67]. The adaptive immune system is essential in clearing SARS-CoV-2, utilizing activated cytotoxic T lymphocytes that destroy infected cells and B lymphocytes that produce neutralizing antibodies against virus-specific antigens. A significant characteristic of COVID-19 is blood lymphopenia, leading to reduced numbers of CD4+ T cells, CD8+ T cells, and B cells [68]. The decrease in lymphocyte levels in COVID-19 patients can be attributed to various factors. One reason is the low levels of IFN-I, which hinders the production of the viral material necessary for antigen presentation and the activation of adaptive immunity. Additionally, lymphopenia may occur due to direct infection of T cells by SARS-CoV-2, cytokine-induced lymphocyte apoptosis, and pyroptosis, MAS-related hemophagocytosis, the sequestration of lymphocytes in the lungs or other organs, the reduction of bone marrow hematopoiesis, and the damage to lymphoid organs induced by the virus (pathological alterations such as atrophy of the splenic white pulp and alteration of the structure of the lymph nodes), suggesting that the direct cytotoxicity of SARS-CoV-2 in lymphatic organs may impair the adaptive immune response in COVID-19 [69]. The failure of germinal center formation in the spleen and lymph nodes may explain why some individuals have suboptimal humoral immunity, potentially leading to the risk of reinfection. However, most COVID-19 patients with mild-to-moderate disease have a strong adaptive immune response. This response includes T cells that target antigens from protein S and nucleoprotein/membrane protein, as well as neutralizing antibodies against protein S-derived antigens. These immune responses can persist for months after the initial infection. It is crucial to note that coordinated adaptive immune responses specific to SARS-CoV-2 play a key role in mitigating the severity of the disease [70]. Variations in individuals’ defense mechanisms may account for the differences in disease progression following infection. Inadequate and uncoordinated adaptive immune responses, often linked to aging, can result in a failure to control the disease. This insufficient response can be attributed to “immunosenescence”, a concept involving the age-related decline in immune function, which includes impairments in both innate and adaptive immune responses, such as compromised pathogen recognition. Other age-related factors contributing to this include low-level systemic inflammation in older adults (“inflammasome”), a higher prevalence of comorbidities in this demographic, and varying degrees of frailty [71,72,73,74]. The immune responses to SARS-CoV-2 may vary based on sex, which could contribute to men being more susceptible to the disease. A study examining differences between sexes in immune characteristics found that male patients have a stronger initial immune response, indicated by higher levels of plasma cytokines. On the other hand, female patients show a more effective T-cell activation [75]. Subjects with multiple underlying diseases or a single serious disease are at greater risk of developing a severe form of COVID-19 (cardiovascular disease, arterial hypertension, diabetes, lung disease, neurodegenerative disorders, immunodeficiencies, kidney disease, obesity, and liver damage) [76]. In this regard, ACE2 expression and activity variations between individuals are believed to impact vulnerability to COVID-19 progression. Paradoxically, increased membrane-bound ACE2 may allow SARS-CoV-2 to invade host cells. At the same time, downregulation of ACE2 (due to SARS-CoV-2-induced endocytosis) precipitates tissue damage, (1) decreasing the inactivation of bradykinins with consequent risk of developing angioedema and (2) dysregulating the RAAS failing to convert Ang II into angiotensin (1–7) [77]. Finally, several other host factors are believed to impact the progression of COVID-19, including epigenetic mechanisms, nutritional status, and ABO blood type [78].

### 4.5. Cytokine Storm

The term “cytokine storm” describes overactive immune responses triggered by various factors, including autoimmune diseases, viral infections, and immunotherapy [79,80,81,82]. Cytokine storms destroy pathogens and cause histotoxicity, affecting various organs [83,84]. Cytokine release syndrome (CRS) is a systemic inflammatory syndrome caused by cytokine storms and has previously been observed in individuals infected with SARS-CoV and MERS-CoV viruses. When the body is infected with a virus, certain molecules activate neighboring cells’ antiviral responses and attract cells of the innate and adaptive immune systems, such as natural killer (NK) cells, macrophages, and gamma delta (gd T) cells [85,86,87,88]. Interferon production helps protect neighboring epithelial cells from being infected, while the release of IL-1b and IL-6 from other immune cells leads to the mobilization of neutrophils and T cells. T cell activation or the lysis of immune cells triggers the secretion of IFN-g and TNF-a, which in turn activates immune cells and endothelial cells, creating a positive feedback loop leading to further release of inflammatory cytokines [89]. These inflammatory mediators may contribute to thrombus formation [57]. This process, called immuno-thrombosis, can also enhance cytokine production and has been attributed to the link between thrombin and inflammasome activation and IL-1 production [90]. Vascular endothelial cells are cells that line the blood vessels. These cells are exposed to various immune mediators and cytokines circulating in the blood. Dysfunction in these cells due to cytokine storms can cause coagulation disorders such as capillary leak syndrome, thrombus formation, and even disseminated intravascular coagulation (D.I.C.). This dysfunction is due to a connection between the immune response and the blood clotting system [57,91]. Cytokine storms inhibit further viral replication and cause secondary tissue damage by secreting large amounts of active mediators and inflammatory factors [57,92,93,94,95]. Inhibition of this self-reinforcing inflammatory cascade may impair viral clearance and inhibit tissue damage. In the COVID-19 study, Huang et al. discovered that individuals admitted to intensive care units (ICUs) had elevated levels of the inflammatory cytokines IL-2, IL-7, IL-10, G-CSF (granulocyte colony-stimulating factor), IFN-γ, M.C.P., and TNF-α in their plasma compared to non-ICU patients.

High levels of cytokines were noted [96]. These cytokines indicated the presence of both the Th1 response and Th2 response in COVID-19. In addition, monocyte activation may indicate that the cytokine storm in COVID-19 is closely related to the imbalance between innate and adaptive immunity. A recent study found that patients with severe COVID-19 had significantly higher levels of IL-6 compared to those with mild or moderate cases. Additionally, patients with severe COVID-19 showed decreased levels of CD4+ T cells, CD8+ T cells, and NK cells, indicating immunosuppression [93].

On the other hand, T lymphocyte cells may be overactivated during cytokine storms in COVID-19 patients, resulting in severe immune dysfunction [97]. A recent systematic review, based on autopsy findings, found fibrin clots with increased CD61-positive platelets and megakaryocytes in the anterior and posterior capillaries. This was observed without complete lumen obstruction in lung and other organ samples from COVID-19 patients [98]. As a result, cytokine storms can directly damage the pulmonary capillary mucosa, leading to alveolar edema and further diffusion of inflammatory cytokines, which damages alveolar structures and impairs pulmonary ventilation [98,99]. Similarly, cytokine storms are associated with the order and severity of organ dysfunction in multiple organ dysfunction syndrome (MODS). Therefore, cytokine storms are considered a crucial factor in determining the outcome of patients with COVID-19 multiorgan pathology.

This study aimed to evaluate the safety and effectiveness of administering high-dose vitamin C (as an add-on to the standard of care) to a population of patients with COVID-19 at different stages of the disease. The current findings suggest that high-dose vitamin C—10 g in 250 cc of saline solution in slow infusion (60 drops/min)—for three consecutive days was safe and associated with shorter hospitalization in patients suffering from COVID-19.

The assumption is based on the observation that ascorbic acid can directly reduce the production of reactive oxygen species (R.O.S.), maintain endothelial barrier function, promote vasodilation, and downregulate the expression of various proinflammatory markers [100]. Some studies have highlighted that vitamin C diminishes the production of chemokines and cytokines such as IL1, IL6, IL8, and TNFα, thus counteracting the inflammatory alterations underlying the lung damage caused by sepsis; this was associated with significantly lower mortality in severely ill patients with pneumonia [32,101]. Vitamin C accumulates in neutrophils, enhancing their chemotaxis, phagocytosis, and microbial killing. It also plays a vital role in apoptosis and the clearance of spent neutrophils from infection sites by macrophages, thereby reducing necrosis and potential tissue damage. Furthermore, vitamin C boosts the development and growth of B- and T-cells, possibly due to its gene-regulating properties [46]. Vitamin C has been widely used in sepsis and ARDS [28,102]. Severe inflammation and cytokine storms contribute to severe ARDS and subsequent mortality in COVID-19 [103]. Considering these effects of the cytokine storm and the close correlation with the prognosis in COVID-19 patients, treatment with high-dose vitamin C was also evaluated, associated with a reduction in C-reactive protein (C.R.P.) levels, procalcitonin (P.C.T.), Interleukin 8 (IL8), and attenuating pulmonary and systemic inflammation. A pilot study conducted in China by Jing Zhang et al. [48] with the use of high-dose vitamin C (24 g/day) in COVID-19 patients with critical illness, although it did not lead to any results in what was the primary end-point (ventilator-free days invasive mechanics), has, however, shown benefits in terms of improvement of the partial pressure ratio of O_2_/inspiratory fraction of O_2_ (P/F) and the safety of using the drug. A retrospective cohort study conducted by D. Gao et al. [104] suggested a benefit in terms of reduced mortality and improved oxygenation status in COVID-19 patients with the use of high-dose vitamin C. Multiple studies have demonstrated the beneficial effects of vitamin C in lowering mortality and reducing hospital stays for patients with non-COVID-related sepsis and ARDS. The CITRIS-ALI study was a significant clinical trial that included 167 patients with ARDS. It was a randomized, double-blind, placebo-controlled trial. Patients were randomly assigned to receive 50 mg/kg of vitamin C every 6 h for four days. This treatment resulted in a statistically significant decrease in 28-day all-cause mortality compared to the placebo [32]. In patients who received a total of 200 mg/kg/day of high-dose intravenous vitamin C (HDIVC) for four days (administered at 50 mg/kg/dose, every 6 h), scores for organ failure were significantly lower than those receiving the placebo and even lower than patients who received lower doses of intravenous vitamin C (50 mg/kg/day administered at 12.5 mg/kg/dose, every 6 h for four days). In another study by Jamali Moghadam Siahkali et al. [105], a dosage of 1.5 g of vitamin C given intravenously every 6 h for five days was used. In addition, improvements in peripheral oxygen saturation and body temperature were found in both groups during hospitalization. The study did not, however, find a statistically significant difference in the reduction in-hospital mortality of patients with moderate and severe disease in the intervention group compared to the placebo, and the main reason could lie in the dosage of intravenous vitamin C used (lower than to other studies conducted). A recent systematic review and meta-analysis of randomized controlled trials and trial sequential analysis found that intravenous vitamin C monotherapy may have mortality benefits for critically ill patients, especially for those at high risk of death. However, the certainty of the evidence available is low [106].

In our study, we administered 10 g of vitamin C every 24 h for three consecutive days, according to our therapeutic protocol, “*Use of Ascorbic Acid in patients with COVID-19*”, developed by our center and registered in March 2020 on the platform *Clinicaltrials.gov*. We aimed to evaluate the safety of administering vitamin C; the treatment was compared to the standard of care alone. The aim of the study was fully achieved, and there was a clear tendency towards the effectiveness of endovenous acid ascorbic treatment even if the statistical significance was not reached. In our study, we did not record any adverse events and we did not record an increase in mortality. Mortality, which we consider an essential safety signal, correcting for all the confounding variables, suggested the effectiveness of vitamin C even if statistical significance was not reached. In addition, a statistical reduction in the length of stay in the hospital was reached. It is worth outlining that our study has some limitations, such as the heterogeneity of the population (presence of patients with different disease stages) or the tendency to use the treatment mainly on patients with more severe forms of the disease. This selection bias could explain the absence of statistically significant effectiveness. Another critical limitation lies in the fact that our sample size is small. The sample size calculated at the beginning of the study was not reached, and this fact could affect the negative results about vitamin C’s effectiveness because the small sample size increases the probability of false negatives. However, according to our results, we have a reasonable certainty about safety.

Finally, the total daily dose of vitamin C used in our study was moderate compared to studies on the effectiveness of intravenous vitamin C in COVID-19 for four to seven days of intravenous vitamin C. A more extended treatment could be necessary to reach effectiveness in infective diseases.

## 5. Conclusions

According to our results, high-dose vitamin C administration showed an excellent short-term safety profile in moderate and severe COVID-19 patients. The infusion of vitamin C is statistically significantly associated with reduced hospital stay. Moreover, high-dose intravenous vitamin C may reduce inflammatory reactions, improve oxygen support, and decrease mortality in COVID-19 patients and other inflammatory diseases without adverse events. It is therefore indicated as a promising therapy for patients with moderate to severe COVID-19, but also for other pathologies in which the hyperinflammatory and inflammasome state play a crucial role in the pathogenesis. More research is necessary to validate these encouraging findings and better understand the role of intravenous vitamin C in treating COVID-19 and other inflammatory conditions. Furthermore, since it is an inexpensive and widely available drug, its possible application and usefulness could represent a change of direction in the treatment strategy for this kind of patient and a large number of inpatients, even to blunt the inflammatory state.

## Figures and Tables

**Table 1:** Clinical variables of the analyzed population.

N	146
Patients undergoing Intravenous High-Dose Vitamin C (IHDVC)	104
Patients not undergoing Intravenous High-Dose Vitamin C (IHDVC)	42
Age ^§^	64.3 (54.9–76.0)
Men _(%)_ *	59.6 (51.3–67.2)
Pre-existing comorbidities *	80.4 (73.0–86.0)
Hypertension *	70.0 (61.8–76.9)
Obesity (BMI ≥ 30 Kg/m^2^) *	40.8 (33.0–49.2)
Diabetes mellitus *	35.6 (28.2–43.9)
Chronic ischemic heart disease *	29.3 (22.4–37.4)
Chronic cerebrovascular disease *	29.1 (22.1–36.9)
Chronic renal failure *	14.7 (9.7–21.5)
Neoplasm (active or previous) *	11.1 (6.9–17.5)
Atrial fibrillation *	9.0 (5.3–15.1)
Body temperature at admission *	37.7 (37.1–38.4)
Oxygen support at admission: None *	37 (29.5–45.2)
Oxygen support at admission: Nose cannulas *	10.2 (6.3–16.4)
Oxygen support at admission: Venturi mask *	25.3 (18.9–33.1)
Oxygen support at admission: CPAP *	15.8 (10.7–22.7)
Oxygen support at admission: NIV S/T *	11.0 (6.8–17.2)
Hospitalization days ^§^	19.0 (13.0–29.5)
Admission to intensive care/ICU _(%)_	8.3
Death _(%)_	10.2

* Data reported as means (95% confidence intervals); ^§^ Data reported as medians (Q1–Q3).

**Table 2:** Clinical variables of the analyzed population according to patients undergoing IHDVC categorization.

Variable	Patients Undergoing IHDVC (n = 104)	Patients Not Undergoing IHDVC (n = 42)	*p*
Age ^§^	64 (53–76)	64 (56–76)	0.8086
Men _(%)_	61.5	54.8	0.4500
Pre-existing comorbidities _(%)_	79.6	82.5	0.6960
Hypertension _(%)_	71.8	65.0	0.4230
Obesity (BMI ≥ 30 Kg/m^2^) _(%)_	39.2	45.0	0.5282
Diabetes mellitus _(%)_	35.9	35.0	0.9177
Chronic ischemic heart disease or Chronic cerebrovascular disease _(%)_	29.1	30.0	0.9180
Chronic renal failure _(%)_	13.6	17.5	0.5535
Neoplasm (active or previous) _(%)_	10.7	12.5	0.7566
Oxygen support at admission: None _(%)_	39.4	30.9	0.3372
Oxygen support at admission: Nose cannulas _(%)_	11.5	7.1	0.4284
Oxygen support at admission: Venturi mask _(%)_	23.1	30.9	0.3220
Oxygen support at admission: CPAP _(%)_	12.5	23.8	0.0895
Oxygen support at admission: NIV S/T _(%)_	12.5	7.1	0.3482
Hospitalization days ^§^	18 (13–27)	24 (13–31)	0.1596
Admission to intensive care/ICU _(%)_	9.7	5.0	0.3620
Death _(%)_	8.6	14.3	0.3103

^§^ Data reported as medians (Q1–Q3).

**Table 3:** Laboratory variables, % variation of the analyzed population (signed rank).

Variable	Patients Undergoing High-Dose Vitamin C Treatment (n = 104)	Patients Not Undergoing High-Dose Vitamin C Treatment (n = 42)	*p*
Hemoglobin _(gr/dL)_	−5.2 (−10.9–1.8)	−3.6 (−14.9–4.4)	0.5064
White cells _(cell/uL)_	24.4 (−6.8–75.9)	1.1 (−23.7–52.5)	0.0656
Neutrophils _(cell/uL)_	16.2 (−22.9–81.5)	−18.6 (−40.6–31.8)	0.0126
Linfocytes _(cell/uL)_	43.2 (2.8–99.6)	38.6 (−7.4–125)	0.9161
Platelets _(cell/µL)_	12.1 (−19.4–48.6)	0 (−15.8–27.7)	0.1735
PCR _(mg/dL)_	−83.9 (−95.0–−10.1)	−82.5 (−95.0–−19.2)	0.7957
Procalcitonin (PCT) _(of/L)_	0 (−51.4–0)	0 (−63.1–0)	0.5827
eGFR _(mL/min/m_^2^_)_	7.7 (0.4–24.2)	4.5 (−7.7–31.0)	0.3599
D-Dimer _(ng/mL)_	−2.6 (−50.3–40.7)	−14.1 (−58.6–2.1)	0.2604
Hematocrit _(%)_	−4.1 (−9.6–2.4)	−1.3 (−10.1–4.9)	0.3407
Monocytes _(cell/µL)_	21.6 (−16.0–71.9)	18.7 (−15.0–103.7)	0.9335
Sodium _(mmol/L)_	0 (−2.1–2.2)	1.4 (−0.7–2.9)	0.1853
Glycemia _(mg/dL)_	−11.2 (−29.9–24.5)	−10.2 (−28.8–16.5)	0.9702

Data reported as medians (Q1–Q3).

**Table 4:** Multivariable regression according to length of hospital stay. Only the final model is shown according to the Hosmer–Lemeshow methodology; for the selection of variables, see the statistical analysis section.

Outcome: Length of Hospital Stays		
Variables	Coefficient (I.C. 95%)	*p* Value
Age	0.13 (−0.02–0.28)	0.089
Male sex	1.26 (−3.06–5.59)	0.565
High-dose Vitamin C treatment	−4.95 (−0.21–−9.69)	0.041

## Data Availability

The data presented in this study are available on request from the corresponding author. The data are not publicly available due to privacy.
